# Molecular and transcriptional basis of bidirectional CD4^+^ T cell exhaustion in oropharyngeal squamous cell carcinoma

**DOI:** 10.1002/mco2.572

**Published:** 2024-06-12

**Authors:** Danni Cheng, Ke Qiu, Daibo Li, Minzi Mao, Yufang Rao, Yao Song, Lan Feng, Xiuli Shao, Chuanhuan Jiang, Yan Wang, Li Li, Xuemei Chen, Sisi Wu, Haiyang Wang, Jun Liu, Haopeng Yu, Wei Zhang, Fei Chen, Yu Zhao, Jianjun Ren

**Affiliations:** ^1^ Department of Oto‐Rhino‐Laryngology West China Hospital, Sichuan University Chengdu Sichuan China; ^2^ Research Core Facility West China Hospital Sichuan University Chengdu China; ^3^ Institute of Clinical Pathology West China Hospital Sichuan University Chengdu Sichuan China; ^4^ West China Biomedical Big Data Center West China Hospital Sichuan University Chengdu Sichuan China

**Keywords:** CD4‐positive T‐lymphocytes, head and neck cancer, immune checkpoint inhibitors, T cell exhaustion, tumor microenvironment

## Abstract

Tumor‐infiltrating CD4^+^ T cells orchestrate the adaptive immune response through remarkable plasticity, and the expression patterns of exhaustion‐related inhibitory receptors in these cells differ significantly from those of CD8^+^ T cells. Thus, a better understanding of the molecular basis of CD4^+^ T cell exhaustion and their responses to immune checkpoint blockade (ICB) is required. Here, we integrated multiomics approaches to define the phenotypic and molecular profiles of exhausted CD4^+^ T cells in oropharyngeal squamous cell carcinoma (OPSCC). Two distinct immune‐promoting (Module 1) and immunosuppressive (Module 2) functional modules in tumor‐infiltrating CD4^+^ T cells were identified, and both the immune‐promoting function of Module 1 cells and immunosuppressive function of Module 2 cells were positively associated with their corresponding exhaustion states. Furthermore, the application of ICBs targeting effector CD4^+^ T cells in Module 1 (αPD‐1) and Treg cells in Module 2 (αCTLA‐4) in mouse models could help reinvigorate the effector function of Module 1‐exhausted CD4^+^ T cells and reduce the immunosuppressive function of Module 2‐exhausted CD4^+^ T cells, ultimately promoting OPSCC tumor regression. Taken together, our study provides a crucial cellular basis for the selection of optimal ICB in treating OPSCC.

## Graphical abstract

Tumor‐infiltrating CD4^+^ T cells in OPSCC were categorized into the immune‐promoting module (Module 1) and the immunosuppressive module (Module 2).The functions of Module 1 and Module 2 were positively associated with the T cell exhaustion states.Blocking the exhaustion progression of the bidirectional modules of CD4^+^ T cells alter the functional balance between Module 1 and Module 2 exhausted cells.

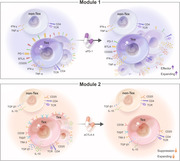

## 1 INTRODUCTION

The establishment of a successful antitumor immune response requires coordination between T cells and various non–T immune cells, constituting a sophisticated intercellular crosstalk network in the tumor microenvironment^1^ (TME). Unlike highly cytotoxic CD8^+^ T cells, CD4^+^ T cells orchestrate the adaptive immune response through remarkable plasticity and play versatile roles, such as facilitating the maturation of antigen‐presenting cells (APCs) as T helper (Th) cells, restricting the effector function of CD8^+^ T cells as regulatory T (Treg) cells, and directly eradicating tumor cells as effector cells.^2^, ^3^

First noted in chronic virus infection, CD8^+^ T cell exhaustion has now been identified as a common phenomenon in the TME of various solid tumors and is characterized by dampened proliferative capacity, poor effector function and high expression of multiple inhibitory receptors.^4^, ^5^ Several studies have been conducted to explore the exhaustion characteristics of CD8^+^ T cells as well as their prognostic significance in head and neck cancers,^6^, ^7^, ^8^ while the exhaustion patterns of CD4^+^ T cells and their associated functional role in the head and neck TME remain unclear. Recently, marked increases in these exhaustion‐suggestive immune checkpoints have also been identified in tumor‐infiltrating CD4^+^ T cells and have been partially linked to disease severity and survival outcomes.^9^, ^10^, ^11^, ^12^ However, their expression patterns differ greatly from those of CD8^+^ T cells, suggesting distinct mechanisms underlying CD4^+^ T cell exhaustion.^13^, ^14^, ^15^ Therefore, functional and transcriptomic correlates are urgently needed, which could help determine whether CD4^+^ T cells acquire exhaustion‐related phenotypes and transcriptomes through an evolutionary process similar to that of CD8^+^ T cells.

Furthermore, the highly heterogeneous function of different CD4^+^ T cell subsets makes it difficult to predict their response to exhaustion‐related signals.^15^ Several recent studies have demonstrated distinct changing patterns of functionality and transcriptomes in the exhaustion‐related process in CD4^+^ T cell subsets, which could differentially respond to certain immune checkpoint inhibitors and achieve controversial antitumor effects.^16^, ^17^, ^18^, ^19^ This finding emphasizes the importance of characterizing the phenotypes and functionality of different exhausted CD4^+^ T cell subsets, based on which selective immunotherapy strategies could be developed to reverse exhaustion in antitumor subsets while enhancing it in protumor subsets.

To address these questions, we combined single‐cell RNA sequencing (scRNA‐seq) and T‐cell receptor sequencing (TCR‐seq) techniques to decipher the multidimensional characterization of tumor‐infiltrating CD4^+^ T cell subsets and monitored their responses to certain immune checkpoint inhibitors, providing a crucial cellular basis for the selection of optimal immune checkpoint blockade (ICB) in treating oropharyngeal squamous cell carcinoma (OPSCC).

## 2 RESULTS

### 2.1 scRNA‐seq of tumor‐infiltrating CD4^+^T cells identified three functional meta‐clusters in human OPSCC

**FIGURE 1:**
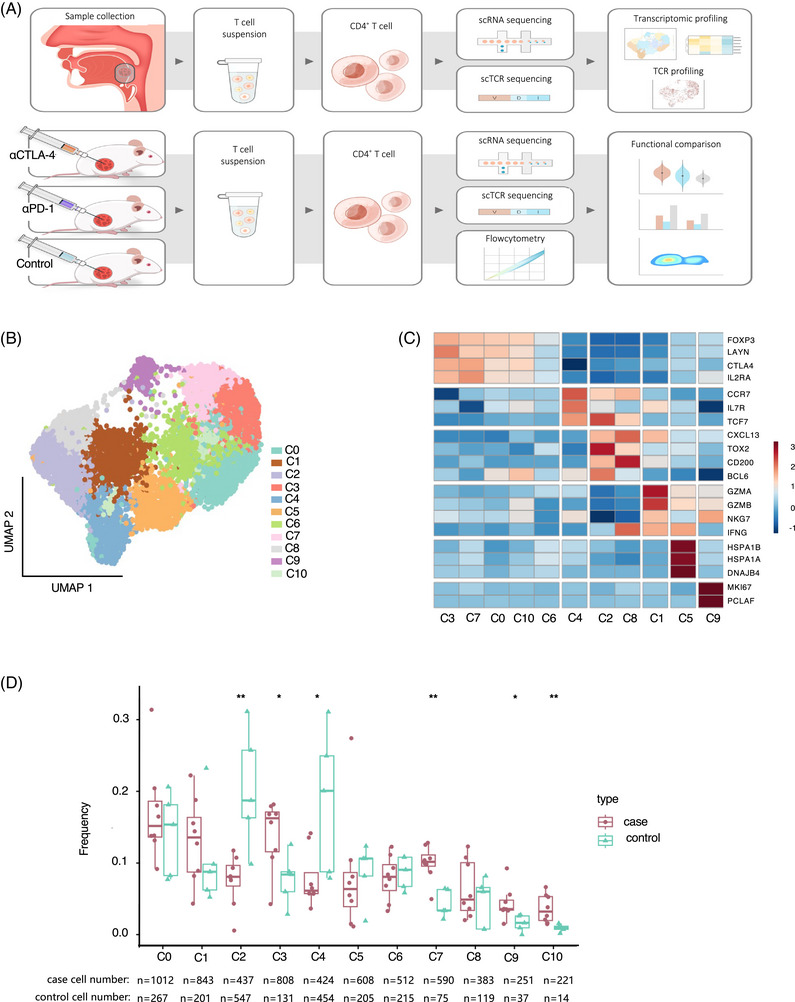
scRNA‐seq of tumor‐infiltrating CD4+T cells identified three functional meta‐clusters in human OPSCC. (A) Schematic of the study design. (B) UMAP plot of CD4^+^ T cells from nine OPSCC cases. Eleven CD4^+^ T cell clusters with different functions were identified in distinct colors. (C) Heatmap of the top transcriptional expressed genes in each CD4^+^ T cell clusters. (D) Bar plot showing the cells proportion of each cluster in case and control samples. Case: tumor tissue (*n* = 8); control, paired adjacent normal tissue (*n* = 5). The cell numbers for each cluster were displayed at the bottom. Statistics were accessed by *t*‐test. *p < 0.05, **p < 0.01.

To decipher the intrinsic heterogeneity within CD4^+^ T cells in the OPSCC TME, we integrated multiomics sequencing data based on scRNA‐seq and scTCR‐seq of eight tumor samples and five adjacent normal samples from nine OPSCC patients (Figure 1A and Table S1). A total of 8354 cells were obtained after quality control, and 11 cell clusters with distinct gene signatures were identified (Figures 1B, C, and S1A). C0, C3, C5, C6, C7, C9, and C10 were characterized by high expression levels of *FOXP3*, representing Treg cells. C5 specifically expresses heat shock protein‐associated genes, including *HSPA1A, HSPA1B*, and *DNAJB*, thus representing heat shock protein‐related Treg cells. C9 displayed high expression of cell cycle‐related marker genes, including *MKI67, TYMS*, and *MCM2*, thus was defined as proliferative Treg cells. C0, C3, C6, C7, and C10 highly expressed *CTLA4* and *IL2RA*, thus representing functionally mature Treg cells. C1 was characterized by the high expression of effector‐like marker genes, including *GZMA, GZMB, NKG7*, and *IFNG*, thus representing effector CD4^+^ T cells. C2 and C8 specifically expressed *CXCL13, TOX2*, and *BCL6* marker genes, which are required for the development and functionality of T follicular helper (Tfh) cells, and thus were defined as Tfh‐like cells. C4 was mainly derived from paratumoral samples (Figure 1C) and displayed high expression of canonical naive marker genes, such as *CCR7* and *IL7R*, consistent with the cell identity of central memory CD4^+^ T cells (Tcm). Except for C4 that harbored cells with a funtionally‐naive status, other clusters could be further categorized into three functional meta‐clusters, namely, Treg cells (C0, C3, C6, C7, and C10), Tfh cells (C2 and C8), and effector cells (C1, C5, and C9) (Figures 1D and S1B).

Collectively, our findings suggest that tumor‐infiltrating CD4^+^ T cells in OPSCC were highly heterogenous and might play versatile roles in tumor progression.

### 2.2 The three functional meta‐clusters of CD4^+^ T cells could be recategorized into two distinct modules based on their associations with exhaustion status

**FIGURE 2:**
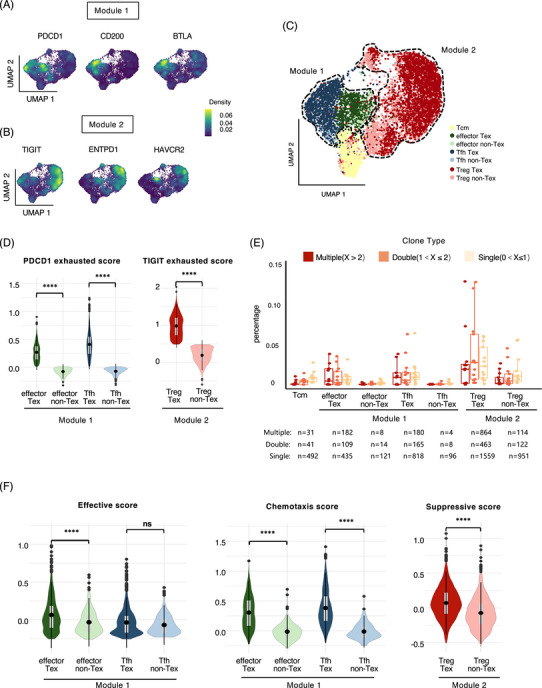
The three functional meta‐clusters of CD4+ T cells could be recategorized into two distinct modules based on their associations with exhaustion status. (A and B) Two panels of inhibitory receptor genes specifically expressed in the Module 1 and Module 2 shown by kernel density estimation. (C) The distribution of CD4^+^ T cells in the Module 1 and Module 2 recategorized by exhaustion levels. non‐Tex, nonexhausted T cell; Tex, exhausted T cell. (D) Comparison of exhaustion scores across subclusters. Module 1 and Module 2 Tex scores were calculated by exhaustion‐related genes specifically expressed in the Module 1 and Module 2, respectively. Data were assessed by the Kruskal–Wallis test. *p < 0.05, **p < 0.01, ***p < 0.001, and ****p < 0.0001. (E) Comparison of TCR clonotype percentages across subclusters. The Module 1 and Module 2 Tex clusters harbored more clonally expanded cells than the Module 1 non‐Tex and Module 2 non‐Tex clusters, respectively. Each Spot represented each sample (*n* = 13). The cell numbers for each cluster were displayed at the bottom. (F) Functional comparison of CD4^+^ Tex and non‐Tex subclusters in Module 1 (effective) and Module 2 (suppressive), respectively. Data were assessed by the Kruskal–Wallis test. *p < 0.05, **p < 0.01, ***p < 0.001, and ****p < 0.0001.

Given the fact that the expression patterns of exhaustion‐related inhibitory receptors in CD4^+^ T cells differ significantly from those of CD8^+^ T cells. To further explore the associations between exhaustion status and the function of different CD4^+^ T cell subclusters, we analyzed the expression patterns of specific functional markers in the CD4^+^ T cells, and we found that multiple well‐known inhibitory receptor genes such as *PDCD1, CD200*, and *BTLA* were the top‐ranked genes in Tfh and effector cells, while *TIGIT, ENTPD1*, and *HAVCR2* were preferentially enriched in Treg cells, which was consistent with the analyses of individual samples (Figures 2A, B and S2A, B). The selective enrichment of these immune checkpoint molecules in the three meta‐clusters may lead to distinct functional characteristics between exhausted (Tex) and nonexhausted T cells (non‐Tex). Therefore, to identify CD4^+^ Tex and non‐Tex subclusters in the three meta‐clusters, we defined cells with high expression of exhaustion‐specific genes as Tex (effector Tex, Tfh Tex, and Treg Tex). And cells with low expression of exhaustion‐specific genes as non‐Tex (effector non‐Tex, Tfh non‐Tex, and Treg non‐Tex) (Figures 2C and D and Table S2).

Interestingly, Treg Tex cells harbored more clonally expanded cells and were more immunosuppressive than Treg non‐Tex, while effector Tex had higher clonal expansion properties and stronger effective scores than their non‐Tex counterparts. Meanwhile, Tfh cells had higher clonal expansion properties and stronger chemotaxis scores than their non‐Tex counterparts. (Figures 2E, F, S3A, and Table S2).

**FIGURE 3:**
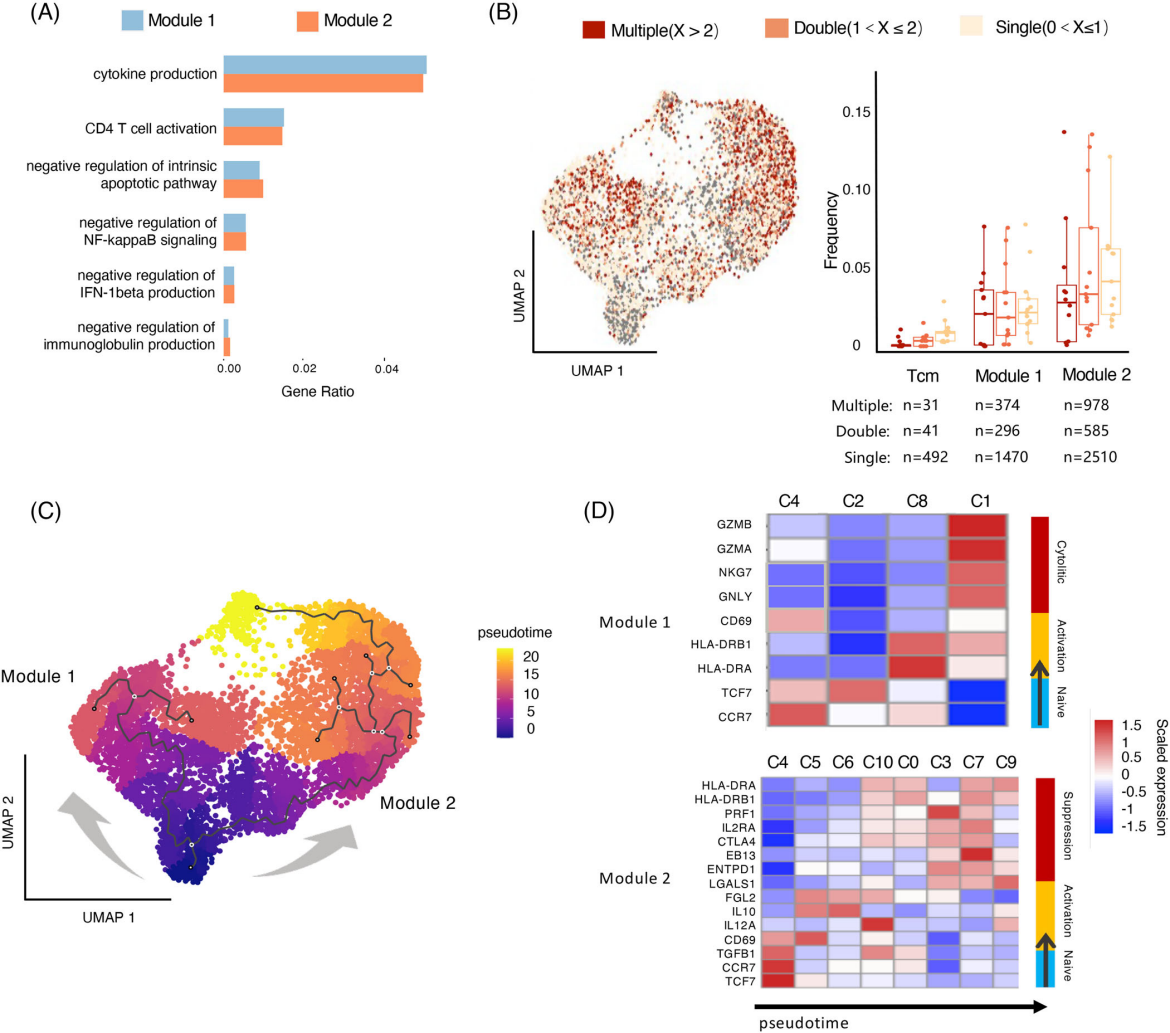
Functional differences between immune‐promoting Module 1 and immunosuppressive Module 2. (A) Significant functional pathways enriched in the Module 1 and Module 2 modules shown by GO terms. (B) Distribution (left) and percentage (right) of the TCR clonotype of CD4^+^ T cells across modules. The clonotype frequency bins were defined as single (0 < *X* ≤ 1), double (1 < *X* ≤ 2) and multiple clonal (*X* > 2) according to their clonotype numbers. Module 2 harbored more clonally expanded cells than Module 1. The cell numbers for each cluster were displayed at the bottom. Data were assessed by the chi‐square test. *p < 0.05, **p < 0.01, ***p < 0.001, and ****p < 0.001. (C) Pseudotime analysis showing two distinct differentiation trajectories corresponding to the Module 1 and Module 2. (D) Pseudotime analysis of gene expression showing dynamic changes along Module 1 and Module 2 differentiation trajectories.

We hypothesized that, when compared with non‐Tex cells, Tex cells would exhibit an activated clonotype expansion state, and effector Tex cells and Tfh Tex cells displayed distinctive functional attributes in contrast to Treg Tex cells. Therefore, these three meta‐clusters were further categorized into two functional modules, in which Tfh and effector cells were divided into immune‐promoting Module 1 and Treg cells were divided into immunosuppressive Module 2. Moreover, functional enrichment analysis demonstrated that the genes specifically expressed by Module 1 cells were mainly enriched in functional pathways related to the activation of both innate and adaptive immune responses, including cytokine production and CD4 T cell activation. In contrast, Module 2 cells were functionally specific for immunosuppression, with their specifically expressed genes enriched in functional pathways, including the negative regulation of intrinsic apoptotic pathway, NF‐kappaB signaling, IFN‐1beta production and immunoglobulin production (Figure 3A). In addition, we found that the Module 2 cells had a higher proportion of multiple clonally expanded cells than the Module 1 (Figure 3B), which might indicate that immunosuppressive functions predominated in these CD4^+^ T cells.

To further investigate their lineage relationships, we performed pseudotime analysis of CD4^+^ T cells based on transcriptional similarities. As expected, two distinct differentiation trajectories were identified for Module 1 and Module 2 cells (Figure 3C). Module 1 cells originated from naive clusters and progressed to effector clusters with gradually enhanced cytotoxicity. Most of Module 2 cells originated from naive clusters and progressed to Treg clusters with gradually augmented immunosuppressive functions (Figure 3D).

Overall, we found two distinct exhaustion patterns between the Module 1 and Module 2 of CD4^+^ T cells and further observed that both the immune‐promoting function of CD4^+^ T cells in the Module 1 and the immunosuppressive function of CD4^+^ T cells in the Module 2 increased with their corresponding exhaustion states.

### 2.3 Functional differences between exhausted and nonexhausted CD4^+^ T cell clusters of two modules according to HPV status

**FIGURE 4:**
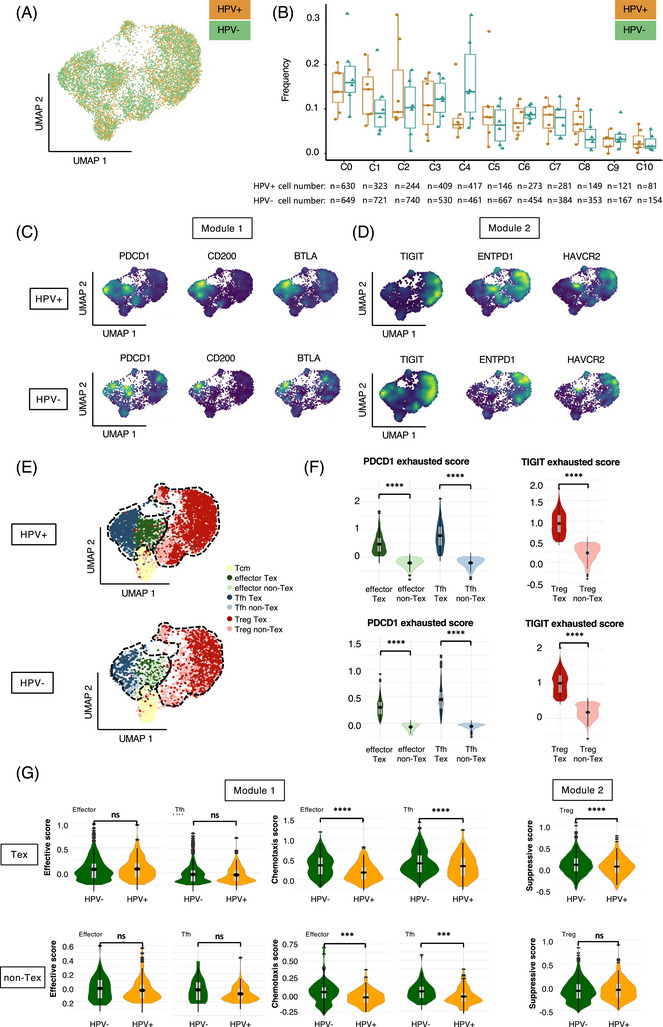
Functional differences between exhausted and nonexhausted CD4^+^ T cell clusters of two modules according to HPV status. (A and B) The distribution (A) and cell counts (B) of CD4^+^ T cell clusters across HPV status (HPV+: *n* = 6, HPV−: *n* = 7). The cell numbers for each cluster were displayed at the bottom. Statistics were accessed by *t*‐test. *p < 0.05, **p < 0.01, ***p < 0.001, and ****p < 0.0001. (C and D) The distribution of inhibitory receptor genes specifically expressed in Module 1 and Module 2 across HPV status, shown by kernel density estimation. (E) The UMAP distribution of CD4^+^ Tex and non‐Tex subclusters in Module 1 and Module 2 across HPV status. (F) The comparison of exhaustion score across subclusters by HPV status. Statistics were assessed by Kruskal–Walls. *p < 0.05, **p < 0.01, ***p < 0.001, and ****p < 0.0001. (G) The comparison of effective and suppressive scores between CD4^+^ Tex and non‐Tex subclusters by HPV status. Statistics were assessed by Kruskal–Walls. *p < 0.05, **p < 0.01, ***p < 0.001, and ****p < 0.0001.

HPV status is an important indicator of prognosis and tumor biology in OPSCC patients. We further conducted subgroup analysis based on HPV status (Figures 4A and B). Consistently, we found similar enrichment preferences for inhibitory receptor genes in the modules of the HPV‐positive and HPV‐negative subgroups (Figures 4C and D). To investigate whether the functional characteristics of the CD4**^+^** Tex and non‐Tex subclusters varied in terms of HPV status (Figures 4E and F), we compared the effective and suppressive scores across the Tex and non‐Tex subclusters in the Module 1 and Module 2 modules, respectively. Within the Module 1, Tfh Tex in HPV‐negative tumors showed significantly higher chemotaxis function than their HPV‐positive counterparts, whereas HPV status seemed to show no influence on the effector function of effector Tex. Meanwhile, within the Module 2, Treg Tex in HPV‐negative tumors showed significantly higher suppressive function than their HPV‐positive counterparts (Figure 4G).

Overall, we found similar expression patterns of inhibitory receptor genes in both HPV‐positive and HPV‐negative tumors, and both suppressive function of Tex in module 2 and chemotaxis function of Tex in module 1 were stronger in HPV‐negative tumors than in their HPV‐positive counterparts.

### 2.4 Flow cytometry analysis validates the functional differences between exhausted and nonexhausted CD4^+^ T cells in the Module 1 and Module 2

**FIGURE 5:**
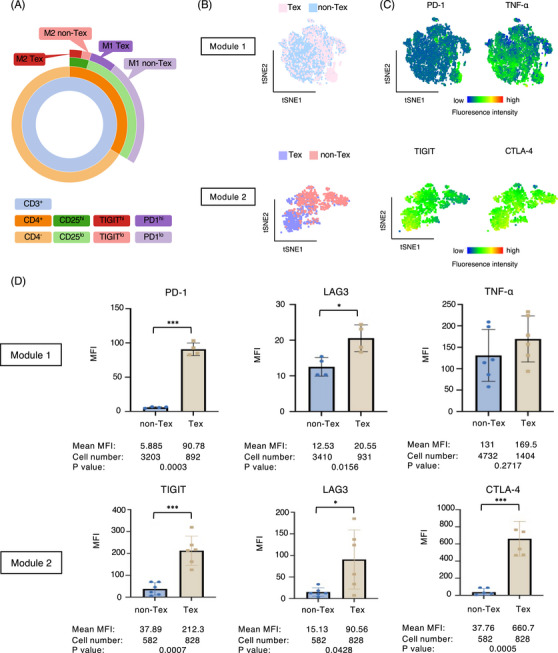
Flow cytometry analysis validates the functional differences between exhausted and nonexhausted CD4^+^ T cells in the Module 1 and Module 2. (A) Gating strategy for sorting CD4^+^ Tex and non‐Tex subclusters. (B) Distribution of Module 1 and Module 2 subclusters projected onto t‐SNE maps using flow cytometry data. (C) t‐SNE heatmaps for specific functional markers applied to Module 1 and Module 2 cell events. (D) The functional differences between CD4^+^ Tex and non‐Tex subclusters in the Module 1 and Module 2 shown by MFI (*n* = 4). Data were shown by the mean ± SEM and were assessed by Student's *t*‐test with Welch's correction. *p < 0.05, **p < 0.01, ***p < 0.001, and ****p < 0.0001. MFI, median fluorescence intensity.

To determine whether the key findings identified at the transcriptional level were also reflected by protein expression, we performed a multiparameter flow cytometry analysis of CD4^+^ T cells isolated from untreated tumor‐bearing mice (Figure S4). We identified the Module 1 Tex, Module 1 non‐Tex, Module 2 Tex, and Module 2 non‐Tex subclusters using appropriate cell surface markers and systematically analyzed their functional differences (Figure 5A). As shown in Figures 5B and C, Module 1 Tex cells were characterized by high expression of PD‐1, whereas Module 2 Tex cells were characterized by high expression of TIGIT, suggesting the functionally exhausted status of Module 1 and Module 2 Tex. Furthermore, though not statistically significant, Module 1 Tex showed a relatively higher expression of TNF‐α, suggesting that Tex may have a higher effector capacity than non‐Tex in Module 1, whereas Module 2 Tex displayed a significantly higher expression of CTLA‐4, a canonical immunosuppressive cytokine (Figure 5D).

Thus, flow cytometry analysis further confirmed that exhausted CD4^+^ T cell subclusters in the two modules were functionally distinct from their nonexhausted counterparts.

### 2.5 Immune checkpoint inhibitors promote tumor regression by altering the functional status of Module 1 and Module 2 exhausted CD4^+^ T cells

**FIGURE 6:**
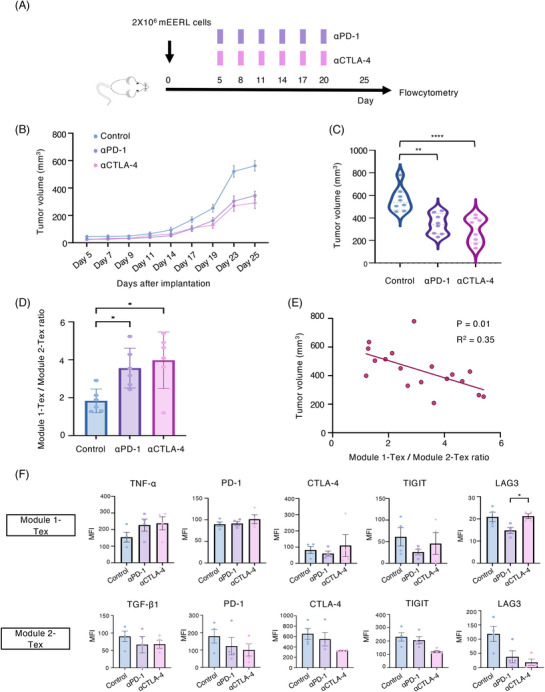
Immune checkpoint blockade promotes tumor regression by altering the functional status of Module 1 and Module 2 exhausted CD4^+^ T cells. (A) Schematic of therapeutic study design. Mice were subcutaneously implanted with the mEERL cell line and monitored for tumor growth every 2−3 days. αPD‐1 (200 µg) or αCTLA‐4 (100 µg) was administered intraperitoneally every 3 days. Tumors were collected on day 25 for subsequent experiments. (B and C) Tumor growth following treatment (*n* = 8), shown by the mean ± SEM. Data were assessed by Tukey's multiple comparison tests. (D) The ratio of Module 1‐Tex cell frequency to Module 2‐Tex cell frequency across treatment groups, shown by the mean ± SEM. Data were assessed by Tukey's multiple comparison tests. (E) Correlation between tumor volume and the ratio of Module 1‐Tex cell frequency to Module 2‐Tex cell frequency on day 25. Correlations were assessed using Pearson correlation coefficients, and *p* < 0.05 and R2 > 0.25 were considered as significantly correlated. (F) Flow cytometry data showing the functional differences in CD4^+^ Tex cells in the Module 1 and Module 2 modules across treatment groups (*n* = 4). Data are shown as the mean ± SEM and were assessed by the Tukey's multiple comparison tests. *p < 0.05, **p < 0.01, ***p < 0.001, and ****p < 0.0001. MFI, median fluorescence intensity.

Previous studies have demonstrated that high expression of inhibitor receptors on T cells provides a potential switch that triggers functional impairment.^20^, ^21^ Therefore, we further examined whether selective ICB could modify the intrinsic function of T cells and improve antitumor immunity (Figure 6A). The results showed that both αPD‐1 and αCTLA‐4 significantly inhibited tumor growth (Figures 6B and C). In addition, both αPD‐1 and αCTLA‐4 significantly increased the ratio of Module 1 to Module 2 Tex, suggesting a shift toward an antitumor phenotype (Figure 6D). We then assessed the correlation between the ratio of Module 1 Tex to Module 2 Tex and tumor growth and found that a higher ratio was significantly correlated with better antitumor effects (Figure 6E). To further demonstrate the specific response of CD4^+^ T cell subclusters to ICB, we systematically analyzed the functional alterations of exhausted and nonexhausted CD4^+^ T cell subclusters after different treatments. As shown in Figure 6F, PD‐1 blockade simultaneously reduced the degree of exhaustion and restored the effector function of the Module 1 Tex cells. However, CTLA‐4 blockade reduced the degree of exhaustion and inhibited the immunosuppressive function of the Module 2 Tex cells.

Thus, selective ICB could significantly promote tumor regression, which might be mediated by its capacity to shift the functional balance between Module 1 and Module 2 Tex cells.

## 3 DISCUSSION

Although CD8^+^ T cells have long been regarded as the main executors of antitumor effects, the dual role of CD4^+^ T cells (immune‐promoting and immunosuppressive) in the TME has recently been established and plays an important role in tumor progression.^22^ Here, we systematically characterized tumor‐infiltrating CD4^+^ T cells from surgically resected human OPSCC and murine tumor models using flow cytometry and single‐cell multiomics sequencing. Our study provides several new insights into CD4^+^ T cell exhaustion: (1) OPSCC tumor‐infiltrating CD4^+^ T cells polarize toward two distinct immune‐promoting (Module 1) and immunosuppressive (Module 2) functional modules with relatively specific expression preferences for exhaustion‐related molecular patterns. (2) Both the immune‐promoting function of Module 1 cells and immunosuppressive function of Module 2 cells are positively associated with their exhaustion states. (3) Application of ICBs targeting Module 1 (αPD‐1) and Module 2 (αCTLA‐4) exhausted CD4^+^T cells can reinvigorate the effector function of Module 1 exhausted CD4^+^ T cells and reduce the immunosuppressive function of Module 2 exhausted CD4^+^T cells, thereby promoting OPSCC tumor regression.

Molecular and cellular insights into CD4^+^ T cell exhaustion in mice have recently been described, and this state shares similarities with CD8^+^ T cell exhaustion.^23^ A previous animal study has shown that tumor‐specific CD4^+^ T cells could induce tumor regression, whereas CD4^+^ T cells with high expression of inhibitory receptors from recurrent tumors failed to achieve similar effects.^17^ Meanwhile, previous clinical studies have also demonstrated that high expression of certain inhibitory receptors on CD4^+^ T cells may be correlated with worse clinical outcomes in patients with malignancies.^10^, ^11^ These findings strongly indicate the critical role of CD4^+^ T cell exhaustion. However, the key molecular and functional characteristics of tumor‐infiltrating exhausted CD4^+^ T‐cells and whether different CD4^+^ T cell subclusters share similar exhaustion processes remains to be further investigated.

Therefore, in this study, we comprehensively characterized the phenotypes of OPSCC tumor‐infiltrating CD4^+^ T cells, and confirmed the existence of two distinct immune‐promoting (Module 1) and immunosuppressive (Module 2) functional modules. Interestingly, we also identified two distinct exhaustion‐related differentiation trajectories in these two modules, which were positively associated with their intrinsic function, respectively. These findings indicate a bidirectional influence of exhaustion on tumor‐infiltrating CD4^+^ T cells, which is much more sophisticated than we expected and has not been reported previously.

Although CD4^+^ T cells also express inhibitory receptors in the setting of chronic infection and tumors, their expression patterns differ greatly between CD4^+^ and CD8^+^ T cells, suggesting different responses to ICB.^14^, ^15^, ^16^, ^17^, ^18^, ^19^, ^20^, ^21^, ^22^, ^23^, ^24^ Several recent studies have shown that PD‐1/PD‐L1 blockade can significantly reduce the expression of inhibitory receptors on conventional CD4^+^ T cells, restore their helper activity, and induce durable tumor control, which is consistent with the findings of this study.^25^, ^26^ However, some studies have demonstrated that combination therapy of PD‐1 blockade with CTLA‐4 blockade, rather than PD‐1 blockade alone, could restore the effector function of Th1‐like CD4^+^ T cells.^27^ This disparity might be associated with the distinct responses of exhausted CD4^+^ T cell subclusters to ICB. Therefore, to systematically characterize the functional influence of different ICB on exhausted CD4^+^ T cell subclusters is necessary. In this case, our study is the first to examine their responses to different ICBs in which PD‐1 blockade and CTLA‐4 blockade can increase the immune‐promoting function of Module 1 cells and suppress the immunosuppressive function of Module 2 cells to promote tumor regression. These findings provide cellular basis for the rational selection of ICBs.

This study analyzes the distinct features of CD4^+^ T cell exhaustion within the TME and elucidates the mechanism of action of immune checkpoint inhibitors in reactivating exhausted CD4^+^ T cells to restore immune response balance and eradicate tumors. This provides a basis for the development of new CD4^+^ T cell‐targeted strategies.

In conclusion, our findings suggest that tumor‐infiltrating CD4^+^ T cells in OPSCC could be categorized into the immune‐promoting module (Module 1) and the immunosuppressive module (Module 2) of which the functions were positively associated with the T cell exhaustion states. Moreover, blocking the exhaustion progression of the bidirectional modules of CD4^+^ T cells can alter the functional balance between Module 1 and Module 2 exhausted cells, leading to overall tumor regression effects. Our study provides a crucial cellular basis for the development of novel CD4^+^ T cell exhaustion‐targeted immunotherapy strategies that should simultaneously restore the immune‐promoting function of antitumor subsets and dampen the immunosuppressive function of protumor subsets.

## 4 MATERIALS AND METHODS

### 4.1 Sample handling

This study was approved by the Ethics Committee of the West China Hospital of Sichuan University (approval number:2021‐908). All patients provided written informed consent, and their clinical characteristics are summarized in Table S1. Fresh tumor tissues (*n* = 8) and paired adjacent normal tissues (*n* = 5) were collected from nine patients with OPSCC who received surgery at West China Hospital of Sichuan University between December 2019 and October 2020. The adjacent normal tissues were away from the tumor margin, and microscopic examination confirmed the absence of tumor cell infiltration. Harvested tissues were cleaned with 1× PBS, and necrotic regions were carefully removed. The tissues were cut into 2−4 mm pieces with sterilized sharp scissors and stored in 1% penicillin streptomycin (HyClone; SV30010) with 1× DMEM (HyClone; SH30243.01) mix. The remaining tissues used for the HPV test were stored in liquid nitrogen and transferred to a −80°C freezer.

### 4.2 Single‐cell suspension preparation

To prepare single‐cell suspensions, all minced tissue samples were transferred into a C tube (Miltenyi; 130‐093‐237) with a tumor dissociation kit (Miltenyi; 130‐095‐929) running the gentleMACS program 37C_h_TDK_3 in the tissue dissociator (Miltenyi). After cell isolation, the tissues were filtered through 40 µm cell strainers to eliminate undissociated tissue clumps and then sorted using human CD3 MicroBeads (Miltenyi; 130‐050‐101) to obtain CD3^+^ T cells. The final sorted CD3^+^T cells were resuspended in HBSS (Thermo‐Gibco; 14175095) and 0.04% bovine serum albumin (Beyotime; ST023‐50 g), and cell viability was tested with a TC20 automatic cell counter (Bio‐Rad) to ensure that cell viability was greater than 80% for subsequent single‐cell sequencing.

### 4.3 Human papillomavirus testing

To extract HPV DNA from tumor tissues, we followed the instructions of the DNA extraction kit (Tiangen; DP304‐02), ground the tumor tissues preserved in liquid nitrogen into powder, added them to a mixture of GD buffer and PW rinsing solution, and mixed them thoroughly. We then dissociated the tissues with Proteinase K reagent and eluted the dissociated tissues sequentially with GB buffer, anhydrous ethanol, and GD buffer. The extracted DNA solution was transferred to a droplet digital PCR (Bio‐Rad) generator for PCR amplification and HPV16 DNA quantitative analysis.

### 4.4 10x Genomics scRNA sequencing and T cell repertoire profiling

scRNA‐seq and TCR‐seq were performed on the NovaSeq 6000 (Illumina) platform via 10× Genomics (10× Genomics; San Francisco, CA, USA). The single‐cell suspension was encapsulated in one lane of the 10× chromium instrument, and libraries were constructed with a Chromium Single Cell 5′ Library & Gel Bead Kit (PN‐1000006), Chromium Single Cell 5′ Library Construction Kit (PN‐1000020), or Chromium Single Cell V(D)J Enrichment Kit (Human T Cell, PN‐1000005) following the standard 10x Genomics protocol (10× Genomics). Library sequencing was performed using a high‐throughput, paired‐end, dual‐indexing pattern, and the sequencing depth was set to a minimum of 20,000 read pairs per cell.

### 4.5 Quality control and preprocessing sequencing data

Following sequencing, Cell Ranger^28^ (version 3.0.2) was used to align the reads with the human reference genome GRCh38 and to generate feature barcode matrices. After creating the matrices, cell‐level filtering was performed using the Seurat R package^29^ (version 4.0, R 4.2.0). Empty droplets, doublets, and cells with more than 5% mitochondrial genes were removed. Following matrix normalization with Seurat default parameters, 2000 variable genes were identified through the FindVariableGenes function and the top 20 principal components of the matrices were determined using principal component analysis. After reducing the dimensionality of the dataset using uniform manifold approximation and projection (UMAP), we identified clusters based on the expression of marker genes. Differentially expressed genes (DEGs) characterized by clustering were determined using the FindAllMarkers function, and DEGs with adjusted *p* values less than 0.05 were considered significant. CD3^+^CD4^+^ T cells based on the expression of CD4 for subsequent analyses.

### 4.6 scRNA‐seq data visualization

The distribution of functional cell subtypes was presented in a two‐dimensional UMAP plot using the Dimplot function of Seurat. Marker gene features were visualized based on gene‐weighted density estimation using Nebulosa^30^ (version 1.3.0, R package). Hierarchical clustering and heatmap generation were performed for single cells based on the normalized expression values of marker genes curated from the literature or from identified significant DEGs and the visualization was accomplished using ggplot2 (version 3.5.0, R package).

### 4.7 Pseudotime trajectory analysis

Pseudotime trajectories were constructed with the Monocle3^31^ (version 3.1.0.0, R package). According to the default parameters of Monocle3, we imported the expression matrices containing all CD4^+^ T cells to construct a single object, extracted the characteristic features of CD4^+^ T cell types, fitted differences within each cell cluster, sorted cell subsets based on DEGs, and constructed a time‐series expression profile. The differentiation trajectories of CD4^+^ T cells were projected onto a UMAP map.

### 4.8 Preprocessing and analysis of scTCR‐seq data

TCR sequence data from chromium single‐cell 5′ RNA‐seq libraries were processed using CellRanger VDJ (version 6.1.1) with default parameters to generate a per‐cell TCR expression matrix. We integrated the TCR immune repertoire data with single‐cell transcriptome data to analyze the dynamic changes in clonotypes in different cell clusters using scRepertoire^32^ (version 1.3.2, R package). We used the absolute frequency of V(D)J gene pairs to define clonotype groups, which were classified as follows: multiple expansion refers to the number of cells of the corresponding clonotypes greater than two (*X* > 2), double expansion refers to cells with two identical clonotypes (1 < *X* ≤ 2), and single expansion means that only one cell expressed a specific clonotype (0 < *X* ≤ 1). The chi‐square test was used to compare the cells of different clonal proliferation types between the two groups, and *p* < 0.05 indicated statistical significance.

### 4.9 Identification of feature modules

To detect hidden functional patterns within CD4^+^ T cells, a gene barcode matrix based on the top 20 DEGs in each cell cluster was constructed, and the matrix was reordered using variable reordering algorithms in the corrplot package (version 0.92, R package).

### 4.10 Definition of function characteristic scores and exhausted cells

To calculate the functional scores of different cell clusters, the AddModuleScore function in Seurat was used to calculate the difference in average expression between the target functional feature genes and background control genes at the single‐cell level. PDCD1 and TIGIT are two of the most canonical exhaustion‐related inhibitory receptor genes, and the usage of PDCD1/TIGIT‐correlated genes for the calculation of exhaustion score has been previous reported.^33^, ^34^ Since *PDCD1* gene was specifically highly expressed in Module 1, the top 30 genes with largest Pearson correlation with *PDCD1* were used to calculate the exhaustion score for Module 1. Likewise, *TIGIT* gene was predominantly expressed in Module 2, and the top 30 genes highly related with *TIGIT* were used to calculate the exhaustion score for Module 2. Cells with exhaustion scores higher than the average exhaustion score were defined as exhausted, whereas the others were defined as nonexhausted cells. Moreover, the *TGFB1, IL10, IL12A, CTLA4, IKZF4, LGALS3, ENTPD1*, and *NT5E* genes representing T cell suppressive function^5^ were used to calculate the suppressive score. The *IL17A, CD69, IFNG, IL4, IL5, IL13, IL17F, TNFRSF8, PALLD*, and *TNF* genes, which symbolize the function of effective T cells,^35^, ^36^ were selected for calculating the effective score. Similarly, CXCL13 is one of the most important chemokines secreted by TFH and CXCL13‐correlated genes could properly reflect the function of TFH.^37^ Therefore, the top 30 genes related with *CXCL13* (the most representative chemokine released by Tfh) were used to calculate the chemotaxis score.^38^ Student's *t*‐test was used to compare the means between two groups, and the Kruskal–Wallis test was used to compare the means of multiple groups. Statistical significance was set at *p* < 0.05.

### 4.11 Gene ontology enrichment analysis

Gene ontology (GO) enrichment analysis was applied by using clusterProfiler^39^ (version 3.15, R package) to detect the gene‐related functional process, and the “goplot” function was used for GO visualization. Signaling pathways with a threshold of ‘’*p* value cutoff = 0.05″ were selected.

### 4.12 Mice

Male C57Bl/6J mice (6–8 weeks) were purchased from Byrness Weil Biotech Ltd. (Chongqing, China) and maintained in a pathogen‐free environment (20 ± 4°C ambient temperature with a relative humidity of 60 ± 5% and a 12‐h light/dark cycle).

### 4.13 Tumor cell line

The mEERL cell line was derived from mouse tonsil epithelial cells expressing HPV‐16 E6, E7, and Ras genes and was purchased from Applied Biological Materials (Richmond, Canada). The mEERL cells were grown in Prigrow IV medium (Cat. No. TM004), comprised of 10% fetal bovine serum, 0.5 µg/mL hydrocortisone, 5 µg/mL transferrin, 5 µg/mL insulin, 1.36 ng/mL triiodothyronine, 5 ng/mL epidermal growth factor, and 1% penicillin/streptomycin solution. Stocks of mEERL were generated upon receipt of the cells and were used for tumor experiments. Cells were tested regularly for *Mycoplasma* contamination, and none tested positive throughout the study.

### 4.14 Tumor implantation

Mice were subcutaneously injected with 2 × 106 mEERL cells in 100 µL of PBS in the right flank. Tumors were measured every 2−3 days using digital calipers. The tumor volume was estimated using the following formula: tumor volume = *Π*/6 × length × width.^2^ Animals were euthanized when tumors surpassed 1000 mm^3^ or ulceration was noted.

### 4.15 In vivo treatments

Tumor‐bearing mice were treated intraperitoneally with αPD1 (rat IgG2a, k, clone 29F.1A12; BioXcell; 200 µg per dose) or αCTLA4 (Syrian Hamster IgG, clone 9H10; BioXcell; 100 µg per dose) every 3 days starting on day 5.

### 4.16 Flow cytometry

The tumors were collected and cut into small pieces (2−4 mm^3^). Samples were then dissociated into a single‐cell suspension using a Tumor Dissociation Kit (Miltenyi Biotec), according to the manufacturer's recommendations. For CD4^+^ T cell analysis, cells were assessed for viability in a LIVE/DEAD Fixable Viability Stain kit (BD Biosciences) for 30 min at 4°C. The samples were washed, stimulated with the Cell Stimulation Cocktail (BioLegend), and blocked with anti‐CD16/CD32 (BioLegend). Cells were simultaneously stained with the following surface antibodies: CD3 (clone 17A2), CD4 (clone GK1.5), CD25 (clone PC61), TGF‐β1 (clone TW7‐16B4), PD‐1 (clone 29F.1A12), CTLA‐4 (clone UC10‐4B9), TIGIT (clone 1G9), and LAG3 (clone C9B7W) purchased from BioLegend, and CD45 (clone 30‐F11) purchased from BD Biosciences. After incubation at 4°C for 30 min, the cells were washed twice with cell staining buffer, fixed, and permeabilized using the Transcription Factor Buffer Set (BD Biosciences). The fixed cells were washed and intracellularly stained with an optimum concentration of TNF‐α (clone MP6‐XT22; BioLegend) and IL‐10 (clone JES5‐16E3; BioLegend) for 50 min at 4°C in the dark. After two more washes, the cells were resuspended in PBS and analyzed using a BD FACSAria SORP Flow Cytometer. Data were analyzed using FlowJo v.10.8.1 (BD Biosciences). Fluorescence minus one (FMO) controls were included to help determine where the gates should be set. The GraphPad Prism (version 9.4.1) software was used for visualization.

### 4.17 Dimension reduction and clustering of multiparameter flow cytometry

To analyze the phenotypes of CD4^+^ T cell subsets, flow cytometry data of CD4^+^ T cells after different treatments were concatenated and processed for TSNE plugins^24^ (iterations = 1000, perplexity = 30, and learning rate = 24) using the parameters CD45 BUV395, CD3 AF700, CD4 APC FIRE 810, CD25 BV711, PD‐1 BV785, CTLA‐4 APC, TIGIT BV421, TNF‐α BV510, TGF‐β1 PE/Dazzle™ 594, IL‐10 PE, and LAG3 BV650 in FlowJo v.10.8.1 (BD Biosciences).

### 4.18 Statistical analysis

Statistical analyses were performed using the R software (Version 3.6.3). The chi‐squared test was used to compare the proportion of TCR clonotype frequencies. The Kruskal–Wallis test was used to compare the single‐cell functional scores. The Student's *t*‐test with Welch's correction was used to compare the median fluorescence intensity between the two cell clusters. Tukey's multiple comparison test was used to compare tumor volumes between the different treatment groups. Statistical significance was set at *p* < 0.05.

## AUTHOR CONTRIBUTIONS

Danni Cheng, Ke Qiu, Daibo Li, Minzi Mao, Yufang Rao, Fei Chen, Yu Zhao, and Jianjun Ren conceptualized the study, designed the experiments, and supervised the project. Danni Cheng, Ke Qiu, and Daibo Li conducted data collection and analysis. Danni Cheng, Ke Qiu, and Yufang Rao contributed to data interpretation and manuscript writing. Yao Song, Lan Feng, Xiuli Shao, Chuanhuan Jiang, Yan Wang, Li Li, Xuemei Chen, Sisi Wu, Haiyang Wang, Jun Liu, Haopeng Yu, and Wei Zhang provided critical revisions and intellectual input. All authors reviewed and approved the final version of the manuscript for submission.

## CONFLICT OF INTEREST STATEMENT

All authors declare no financial or nonfinancial conflict of interest.

## ETHICS STATEMENT

All human samples experiments were approved by the Ethics Committee of the West China Hospital of Sichuan University (approval number: 2021−908), and written informed consent was obtained from all participants. And all animal experiments were approved by the Animal Ethics Committee of the West China Hospital (approval number: 20220511001). The experiments complied with the Ethical Guidelines for the Care and Use of Laboratory Animals set by the China Association of Laboratory/Animal Care, and the animals were humanely euthanized at defined endpoints.

## Supporting information

Supporting Informtion

Supporting Informtion

## Data Availability

The datasets used and/or analyzed during the current study are available from the corresponding author on reasonable request.
