# Supplementary material for: Molecular and transcriptional basis of bidirectional CD4+ T cell exhaustion in oropharyngeal squamous cell carcinoma
Source: MedComm (2020). 2024 Jun 12;5(6):e572. doi: 10.1002/mco2.572 (PMC11167179; doi:10.1002/mco2.572)
Supplement: Supplementary file 1 — Supporting Informtion [file MCO2-5-e572-s001.docx]

**Supplementary files**

**Molecular and Transcriptional Basis of Bidirectional CD4^+^ T Cell Exhaustion in Oropharyngeal Squamous Cell Carcinoma**

**Authors:**

Danni Cheng,^1#^ Ke Qiu,^1#^ Daibo Li,^1#^ Minzi Mao,^1#^ Yufang Rao,^1#^ Yao Song,^1^ Lan Feng,^1^ Xiuli Shao,^1^ Chuanhuan Jiang,^1^ Yan Wang,^2^ Li Li,^3^ Xuemei Chen,^2^ Sisi Wu,^2^ Haiyang Wang,^1^ Jun Liu,^1^ Haopeng Yu,^4^ Wei Zhang,^4^ Fei Chen,^1^* Yu Zhao,^1,4^* Jianjun Ren^1,4^*

**Affiliations:**

^1^ Department of Oto-Rhino-Laryngology, and National Clinical Research Center for Geriatrics, West China Hospital, West China Medical School, Sichuan University, Chengdu, Sichuan, 610041, P.R China.

^2^ Research Core Facility of West China Hospital, Sichuan University, Chengdu, 610041, P.R China.

^3^ Institute of Clinical Pathology, West China Hospital, Sichuan University, Chengdu, Sichuan, 610041, P.R China.

^4^ West China Biomedical Big Data Center, West China Hospital, Sichuan University, Chengdu, Sichuan, 610041, P.R China.

^#^ These authors contributed equally to this work

* Corresponding author:

Jianjun Ren, Department of Oto-Rhino-Laryngology, West China Hospital, Sichuan University, Chengdu, Sichuan, 610041, P.R China, Email: [Jianjun.Ren@scu.edu.cn](mailto:Jianjun.Ren@scu.edu.cn)

Yu Zhao, Department of Oto-Rhino-Laryngology, West China Hospital, Sichuan University, Chengdu, Sichuan, 610041, P.R China, Email: [yutzhao@VIP.163.com](mailto:yutzhao@VIP.163.com)

Fei Chen, Department of Oto-Rhino-Laryngology, West China Hospital, Sichuan University, Chengdu, Sichuan, 610041, P.R China, Email: [hxchenfei@163.com](mailto:hxchenfei@163.com)

**Supplementary Figure 1. The supplementary summary of CD4^+^ T cell profiling in Figure 1.**

(A) Cell-type fractions in each sample. (B) Distribution of CD4^+^ T cells across case and control.

**Supplementary Figure 1**. **Supplementary summary of Figure 2A-B.**

The distribution of inhibitory receptor genes specifically expressed in Module 1 and Module 2 in each sample, shown by kernel density estimation.

**Supplementary Figure 3. Supplementary summary of Figure 2.**

(A) Functional comparison of CD4+ Tex and non-Tex subclusters in Module 1 (suppressive) and Module 2 (effective), respectively. Data were assessed by the Kruskal-Wallis test. *P < 0.05, **P < 0.01, ***P < 0.001 and ****P < 0.0001.

**Figure S4. Flowcytometry gating strategy in tumor-bearing mice.**

**Supplementary Table 1. Patient information**

| **Patients number** | **#opc1** | **#opc2** | **#opc3** | **#opc4** | **#opc5** | **#opc6** | **#opc7** | **#opc8** | **#opc9** |
| --- | --- | --- | --- | --- | --- | --- | --- | --- | --- |
| **Age (years)** | 73 | 58 | 52 | 48 | 63 | 68 | 77 | 71 | 64 |
| **Gender** | M | M | F | M | M | M | F | M | M |
| **T** | 4 | 2 | 1 | 3 | 4 | 2 | 2 | 3 | 2 |
| **N** | 2 | 2 | 2c | 2 | X | 2b | 2 | 2c | 1 |
| **M** | 0 | 0 | 0 | 0 | 0 | 0 | 0 | 0 | 0 |
| **Stage** | IV | IV A | IV A | IV A | IV | IV | IV | IV A | III |
| **HPV status** | positive | positive | negative | positive | positive | negative | negative | negative | negative |
| **Sample** | case & control | control | case & control | case | case & control | case | case | case | case & control |
